# Ameliorative Effects of High‐Intensity Interval Training (HIIT) and Methylphenidate Against Tramadol‐Induced Cognitive Impairment: The Role of Hippocampal Oxidative Stress

**DOI:** 10.1002/brb3.70925

**Published:** 2025-09-27

**Authors:** Sara Shirazpour, Farahnaz Taheri, Gholamreza Sepehri, Manzumeh Shamsi Meymandi, Mahla Zangiabadizadeh, Mostafa Zangiabadi, Najmeh Sadat Hosseini, Mohammad Amin Rajizadeh, Sara Sheikhi, Nazanin Sabet

**Affiliations:** ^1^ Physiology Research Center, Institute of Neuropharmacology Kerman University of Medical Sciences Kerman Iran; ^2^ Neurology Research Center, Institute of Neuropharmacology Kerman University of Medical Sciences Kerman Iran; ^3^ Veterinary Medicine Student, School of Veterinary Medicine Shahid Bahonar University of Kerman Kerman Iran; ^4^ Bone Vascular and Microcirculation Laboratory, Department of Kinesiology University of Texas at Arlington Arlington Texas USA

**Keywords:** tramadol, methylphenidate, high‐intensity interval training (HIIT), learning and memory, oxidative stress

## Abstract

**Background:**

Tramadol (TM) abuse results in significant cognitive dysfunction. This study aimed to investigate the impact of an 8‐week high‐intensity interval training (HIIT) regimen and methylphenidate (MPH) administration (alone and in combination) on cognitive function and hippocampal oxidative stress markers following chronic TM administration.

**Methods:**

Fifty‐six adult male rats (200–250 g) were divided into eight groups and received one of the following treatments: tramadol (50 mg/kg, intraperitoneally, 5 days/week for the first month and 3 days/week for the second month), methylphenidate (10 mg/kg, intraperitoneally, 3 times/week for 60 days), HIIT (five sessions/week for 8 weeks), or saline (1 mL, intraperitoneally, daily for 60 days). Learning and memory were assessed using the Morris water maze (MWM) and passive avoidance tests. Hippocampal malondialdehyde (MDA), glutathione peroxidase (GPx), and total antioxidant capacity (TAC) were measured using TBARS and FRAP methods, respectively. Hippocampal nitric oxide (NO) levels were determined with a commercial assay kit.

**Results:**

Tramadol induced significant impairments in learning and memory (*p* < 0.001). MPH, HIIT, and their combination attenuated these deficits. Tramadol and MPH increased MDA and NO levels (*p* < 0.001) and reduced TAC (*p* < 0.001). In contrast, HIIT reduced these parameters, even in the presence of MPH. In fact, HIIT reversed the adverse effects of tramadol and MPH by reducing MDA and NO (*p* < 0.001) and by increasing GPx (*p* < 0.05) and TAC (*p* < 0.001).

**Conclusion:**

Although both MPH and HIIT interventions show promise in mitigating tramadol‐induced cognitive deficits, their mechanisms of action appear to differ significantly. HIIT likely exerts its effects by modulating oxidative stress, whereas MPH seems to disrupt it, suggesting distinct underlying mechanisms. Further studies are required to elucidate these mechanisms in greater detail.

## Graphical abstract

Both MPH and HIIT improved tramadol‐induced cognitive impairments. HIIT improved these disorders, probably through modulating oxidative stress in the hippocampus. Although MPH disrupted the balance of oxidative stress, it probably improved cognitive impairments through another mechanism.

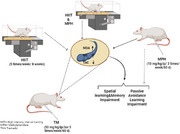

## 1 Introduction

Drug abuse is a major global health concern, with an estimated 35–72 million people affected by drug use disorders (Das and Horton 2019). Tramadol (TM), a synthetic codeine analog, is widely used for pain relief (Subedi et al. 2019) and recreational purposes (Ahmed et al. 2018), particularly among young adults. Chronic TM consumption is associated with neurological side effects (Raj et al. 2019), cognitive dysfunction, and psychological changes (Ahmed et al. 2018; Attoh‐Mensah et al. 2021). Cognitive function—especially learning and memory—is critical for quality of life and longevity (Dyrbye et al. 2017; Kuiper et al. 2017). One major mechanism underlying cognitive impairment is hippocampal redox imbalance (G.‐Y. Choi et al. 2024; Forqani et al. 2023), and chronic TM exposure has been shown to increase oxidative stress in the hippocampus (Khatmi et al. 2022). Thus, protecting against oxidative stress may be essential for preserving cognitive function (Franzoni et al. 2021).

Methylphenidate (MPH), commonly known as Ritalin, is frequently misused by students and young people as a cognitive enhancer (Outram 2010; Lucke et al. 2018). MPH improves learning and memory by enhancing synaptic plasticity via noradrenergic mechanisms (Dommett et al. 2008).

Exercise training is a well‐established non‐pharmacological approach for improving cognitive function (Haynes et al. 2019). It induces structural and cellular changes in the hippocampus that enhance learning and memory (Loprinzi 2019). For example, voluntary exercise attenuates morphine‐induced cognitive deficits via brain‐derived neurotrophic factor (BDNF) signaling (Miladi‐Gorji et al. 2011). Exercise has also been shown to reduce hippocampal reactive oxygen species (ROS) in Alzheimer's disease models (Gomez‐Cabrera et al. 2008; Bouzid et al. 2018). High‐intensity interval training (HIIT), which alternates between short periods of high‐intensity activity (> 85% maximal heart rate) and low‐intensity recovery (∼50% maximal heart rate) (Costigan et al. 2015), produces faster physiological adaptations than other exercise modalities (Leite et al. 2021; Ní Chéilleachair et al. 2017) and has been reported to improve cognitive function and mental health (Leahy et al. 2020).

Tramadol consumption is associated with significant cognitive impairments. Given the established efficacy of MPH in enhancing learning and memory and the protective role of HIIT against cognitive decline, we proposed that these interventions could mitigate tramadol‐induced deficits. While both MPH and HIIT are known to enhance cognitive function, they engage distinct mechanisms. HIIT is postulated to act through anti‐inflammatory (De Oliveira Santos et al. 2021) and antioxidant pathways (Molaei et al. 2020), alongside the upregulation of BDNF (Xu et al. 2021). In contrast, MPH primarily functions by enhancing catecholaminergic signaling in the prefrontal cortex (Di Miceli et al. 2022). We hypothesize that these interventions not only converge on the common outcome of improved cognition but may also exhibit synergistic effects. Specifically, HIIT may create a favorable neural environment by reducing oxidative stress and inflammation, thereby potentiating the cognitive‐enhancing effects of MPH. Therefore, this study aimed to investigate the individual and combined effects of MPH and HIIT on learning and memory impairments in a rat model of tramadol consumption. Furthermore, to explore a potential mechanistic pathway, we assessed the effects of these treatments on hippocampal oxidative stress parameters. Elucidating this interaction is crucial, as it could pave the way for developing more effective and safer combinatorial therapeutic protocols for substance‐induced cognitive disorders.

## 2 Materials and Methods

### 2.1 Animals

Fifty‐six male Wistar rats (200 and 250 g) were obtained from the Animal Center of Kerman University of Medical Sciences (Kerman, Iran). Animals were randomly housed under a 12‐h light/dark cycle at 22 ± 2°C, with ad libitum access to food and water (4 rats per cage; 40 cm × 20 cm × 15 cm). All experimental procedures were approved by the Institutional Animal Ethics Committee of Kerman University of Medical Sciences (Ethics code: IR.KMU.AEC.1401.005).

### 2.2 Drugs

TM (Sigma‐Aldrich, USA) and MPH (Actover Co., Iran) were dissolved in 0.9% saline. TM was administered intraperitoneally (i.p.) at 50 mg/kg and MPH at 10 mg/kg.

### 2.3 Exercise Protocol

The training protocol was adapted from Rezaei et al. (2023). Briefly, before the training experiments, all HIIT groups rats were habituated to the treadmill at 8 m/min speed and 0% inclination, 10–15 min/day, five times a week for 2 weeks (Rezaei et al. 2023). Then the EX, TM + EX, MPH + EX, and TM + MPH + EX groups experienced an increasing running test to define their maximal speed (Vmax). During this test, rats initially ran at a speed of 6 m/min for 2 min. Subsequently, the treadmill speed was increased by 2 m/min every 2 min until the animals reached exhaustion. The highest speed the animals were able to sustain prior to exhaustion was recorded as the maximum running velocity (Vmax) (Rezaei et al. 2023). To maintain appropriate training intensity, Vmax was reassessed every 2 weeks, and the exercise protocol was adjusted accordingly for the following 2‐week training period (Rezaei et al. 2023). The training sessions were conducted five times per week for 8 weeks (Rezaei et al. 2023).

### 2.4 Experimental Protocol

To study the impact of HIIT and MPH on TM‐induced cognitive deficits, rats were randomly allocated into 8 groups (*n* = 7) as follows:

*Group (SAL)*: Rats received saline (1 mL/day for five consecutive times/week in the first month and three consecutive times/week in the second month).

*Group (TM)*: Rats received tramadol (50 mg/kg/day for five consecutive times/week in the first month and three consecutive times/week in the second month).

*Group (MPH)*: Rats received methylphenidate (10 mg/kg/3 consecutive times/week/60 days).

*Group (HIIT)*: Rats underwent HIIT (five consecutive times/week for 8 weeks) and received saline (1 mL/day for five consecutive times/week in the first month and three consecutive times/week in the second month).

*Group (TM + MPH)*: Rats received tramadol (50 mg/kg/day for five consecutive times/week in the first month and three consecutive times/week in the second month) and received methylphenidate (10 mg/kg/three consecutive times/week/60 days).

*Group (TM + HIIT)*: Rats received tramadol (50 mg/kg/d for five consecutive times/week in the first month and three consecutive times/week in the second month) and underwent HIIT (five consecutive times/week for 8 weeks).

*Group (MPH + HIIT)*: Rats received methylphenidate (10 mg/kg/three consecutive times/week/60 days) and underwent HIIT (five consecutive times/week for 8 weeks).

**FIGURE 1:**
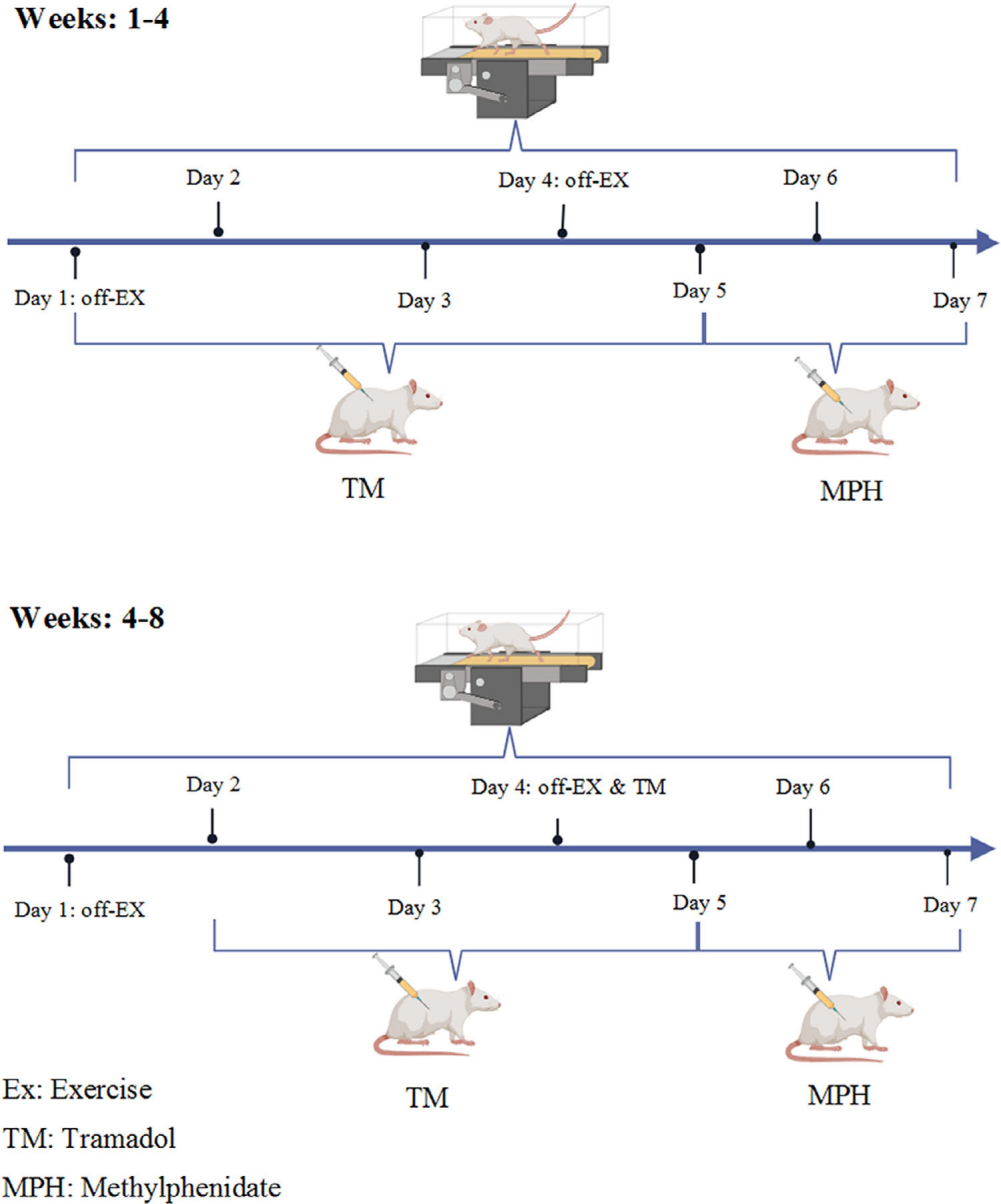
The timeline.

*Group (TM + MPH + HIIT)*: Rats received tramadol (50 mg/kg/day for five consecutive times/week in the first month and three consecutive times/week in the second month) and methylphenidate (10 mg/kg/three consecutive times/week/60 days), and underwent HIIT (five consecutive times/week for 8 weeks). The timeline of treatment is shown in Figure 1. The Morris water maze (MWM) and passive avoidance tasks were carried out separately between 8:00 a.m. and 2:00 p.m. in all groups. During all behavioral testing and subsequent biochemical analysis, the experimenter was blinded to the group allocation of the animals. This was ensured by having a separate researcher code the samples and treatments.

### 2.5 Behavioral Assessments

#### 2.5.1 MWM Test

The MWM test is a widely used paradigm for studying spatial learning and memory in rodents. The apparatus consists of a circular pool with a diameter of 160 cm and a height of 80 cm, filled with water to a depth of 40 cm. The pool is conceptually divided into four equally sized quadrants based on four geographic locations: North (N), South (S), East (E), and West (W).

A square escape platform (10 cm wide) was hidden 1.5 cm beneath the water surface in the center of the northeast quadrant. To provide spatial cues, extra‐maze geometric images were fixed on the walls surrounding the maze, and the room was dimly lit. The performance of the animals was recorded and tracked using a smart video tracking system (Noldus Ethovision XT, version 7.1, the Netherlands). The water temperature was maintained at 25 ± 2°C.

##### 2.5.1.1 Learning Acquisition Phase

To evaluate learning, the animals underwent training across three consecutive blocks. Each block consisted of four sequential trials with an intertrial interval of 30 s. In each trial, which lasted 60 s, rats were released randomly from one of the four designated start locations, always facing the tank wall. The location of the hidden platform remained constant throughout all training blocks.

During a trial, the rat was allowed to swim freely to locate the hidden platform within the 60‐s timeframe. If an animal found the platform, it was permitted to remain on it for 20–30 s before being returned to its home cage for a 20‐ to 30‐s rest period until the next trial began. If a rat failed to find the platform within 60 s, it was gently guided to the platform by the experimenter and allowed to stay there for 20–30 s, followed by the same rest period in its cage. For each trial, the latency (time spent), path length (distance traveled) to find the platform, and swimming velocity were recorded and analyzed.

##### 2.5.1.2 Memory Probe Test

A single probe trial was given 2 h after the last training trial to test the long‐term spatial memory in the water maze. In the probe phase, the platform was removed, and memory retention was tested. The time and distance spent in the target quadrant (the platform quadrant in the training phase) were analyzed to measure spatial memory retention.

##### 2.5.1.3 Visible Platform Test

Following the probe trial, a visible platform test was performed to control for potential deficits in sensory‐motor coordination, visual perception, or motivation. For this test, the platform was raised 2 cm above the water surface and covered with aluminum foil to make it clearly visible. The animal's ability to escape to this visible platform was then evaluated (Taheri et al. 2022; Taheri, Joushi, et al. 2023; Rafie et al. 2025).

#### 2.5.2 Passive Avoidance Test

The passive avoidance task is a fear‐motivated test used to evaluate associative learning and memory in rodents. In this test, the animal learns to associate a previously neutral environment (a dark compartment) with an aversive stimulus (an electric shock) and subsequently avoids it.

##### 2.5.2.1 Apparatus

A shuttle‐box apparatus with dimensions of 100 (L) cm × 25 (W) cm × 25 (H) cm was used. The box consisted of two compartments (one light and one dark), separated by a retractable door.

##### 2.5.2.2 Habituation and Training Protocol

1. *Habituation*: Each animal was first habituated to the apparatus by being placed in the light compartment for 5 min with the door open, allowing it to freely explore both chambers. After this period, the animal was returned to its home cage. This process was repeated once. Any animal that failed to enter the dark compartment during habituation was excluded from the study.
2. *Training*: Two hours after habituation, the training trial was conducted. The animal was placed in the light compartment, and the door was opened after a 10‐s delay. Upon entry into the dark compartment, the animal immediately received a mild electric shock (50 Hz, 0.5 mA, 2 s) delivered through the grid floor. The door was then closed. This procedure was repeated at 2‐min intervals until the animal learned the avoidance response, defined as remaining in the light compartment for 120 consecutive seconds. The number of shocks required to reach this criterion was recorded as a measure of acquisition learning.

##### 2.5.2.3 Memory Retention Test

Twenty‐four hours after the training phase, memory retention was assessed. The animal was placed in the light compartment with the door closed. After a 10‐s delay, the door was opened. The following parameters were recorded:

- *Step‐Through Latency (STL)*: The time taken for the animal to enter the dark compartment with all four paws.
- *Time in Dark*: The total time spent in the dark compartment during a 5‐min period after the door opening (Taheri, Esmaeilpour, et al. 2023).

All behavioral tests were conducted 24 h after the administration of saline, TM, MPH, and HIIT.

### 2.6 Hippocampus Tissue Dissection

After the behavioral tests, the rats were anesthetized with carbon dioxide and then sacrificed. The hippocampi were rapidly removed and dissected. The tissue was homogenized and centrifuged at 14,000 rpm at 4°C for 20 min.

### 2.7 Hippocampus Oxidative Stress Assessment

MDA compound was measured as a lipid peroxidation index. The MDA level was measured by the related kit via the TBARS method (Shirazpour et al. 2025). The TAC level was measured via the FRAP method using spectrophotometry and a specific kit (Hosseini et al. 2024; Benzie and Strain 1996). The GPX activity was measured by a specific related kit (Sadat Hosseini et al. 2025). The Griess method was used to measure the levels of NO in serum (Yucel et al. 2012).

### 2.8 Statistical Analysis

Data analysis was performed using GraphPad Prism software (version 8). The Shapiro–Wilk test was used to assess the normality of the data. A repeated‐measures two‐way analysis of variance (ANOVA) was used for the learning phase of the MWM, while a one‐way ANOVA was applied to analyze the other data. When a statistically significant difference was found between groups, Tukey's multiple comparison test was performed as a post hoc analysis. The significance level was set at *p < 0.05.

## 3 Results

### 3.1 Effects of the MPH and HIIT on MWM Following TM Administration

#### 3.1.1 Effects of the MPH and HIIT on Learning Phase of MWM

**FIGURE 2:**
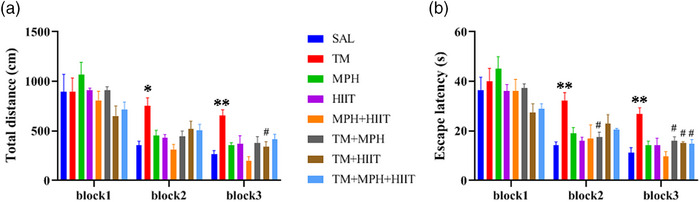
Performance of experimental groups in the MWM (learning phase). (A) Total distance was higher in TM rats compared with SAL, while HIIT reduced distance in the TM + HIIT group in Block 3. (B) Escape latency was increased in TM rats compared with SAL. Both MPH and HIIT reduced escape latency in the TM + MPH, TM + HIIT, and TM + MPH + HIIT groups compared with TM. Data are mean ± SEM (*n* = 7). Two‐way repeated measures ANOVA (A) and one‐way ANOVA (B). *p < 0.05, **p < 0.01 versus SAL; ^#^ *p* < 0.05 versus TM.

Two‐way repeated measures ANOVA showed that the TM group had significantly increased total distance and escape latency in Block 2 (Figure 2A, *p* < 0.05; Figure 2B, *p* < 0.01) and Block 3 (Figure 2A, *p* < 0.01; Figure 2B, *p* < 0.01) compared with the SAL group.

Methylphenidate administration improved spatial learning in tramadol‐exposed rats, as indicated by reduced escape latency in Blocks 2 and 3 (Figure 2B, *p* < 0.05) compared to the TM group. HIIT also enhanced learning, with reduced total distance (Block 3, Figure 2A, *p* < 0.05) and escape latency (Block 3, Figure 2B, *p* < 0.05) compared with TM. Furthermore, the combination of MPH and HIIT significantly decreased escape latency in the TM + MPH + HIIT group compared with TM in Block 3 (Figure 2B, *p* < 0.05).

#### 3.1.2 Effects of the MPH and HIIT on Memory Phase of MWM

**FIGURE 3:**
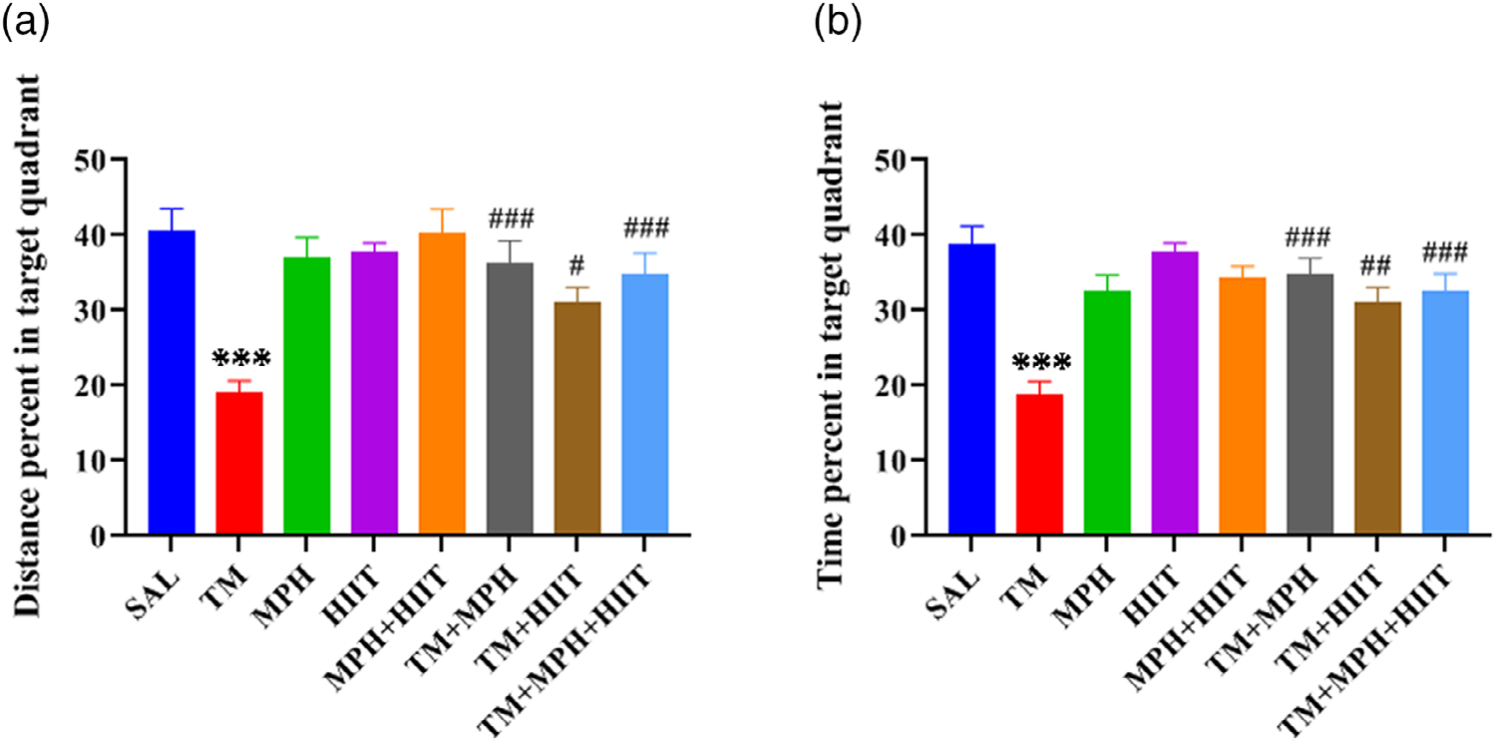
Performance of experimental groups in the MWM (memory phase). (A) Distance percentage in the target quadrant decreased in TM compared with SAL but increased in TM + MPH, TM + HIIT, and TM + MPH + HIIT groups. (B) Time spent in the target quadrant decreased in the TM compared with SAL but increased in the TM + MPH, TM + HIIT, and TM + MPH + HIIT groups. One‐way ANOVA followed by Tukey's test. Data are mean ± SEM (*n* = 7). ***p < 0.001 versus SAL; ^#^ *p* < 0.05, ^##^ *p* < 0.01, ^###^ *p* < 0.001 versus TM.

The probe test was performed 24 h after the acquisition phase to examine long‐term spatial memory retention. The obtained results included the mean percentage (%) for a time as well as distance. The probe test was performed 24 h after the acquisition phase to evaluate long‐term spatial memory. TM rats traveled a shorter distance and spent less time in the target quadrant compared with SAL rats (Figure 3A, *p* < 0.001; Figure 3B, *p* < 0.001), indicating impaired memory.

Methylphenidate prevented this impairment, as TM + MPH rats traveled longer distances and spent more time in the target quadrant compared with TM (Figure 3A, *p* < 0.001; Figure 3B, *p* < 0.001). HIIT also improved memory, with significant increases in distance traveled (Figure 3A, *p* < 0.05) and time spent (Figure 3B, *p* < 0.01) in the target quadrant compared with TM. In addition, the combination of HIIT and methylphenidate was able to increase distance traveled and time spent in the target quadrant in the TM + MPH + HIIT group compared with the TM group in Block 3 (Figure 3A,B, *p* < 0.001).

There was no significant difference in the swimming speed and latency to find the visible platform among the experimental groups. Therefore, our manipulations did not induce any motor or sensory deficits in the experimental animals (Table 1).

**TABLE 1:** (A) Velocity and (B) visible test data. There was no significant difference in swimming speed and latency to find the visible platform among experimental groups.

	A	B
	Velocity (cm/s)	Time to find the platform (s)
Group	Mean ± SEM	Mean ± SEM
SAL	21.01± 0.85	18.83 ± 1.31
TM	20.41 ± 0.81	20.83 ± 2.14
MPH	21.55 ± 0.69	19.55 ± 1.93
HIIT	23.25 ± 0.61	18.58 ± 0.96
TM + MPH	21.71 ± 0.71	21.03 ± 1.98
TM + HIIT	21.52 ± 0.53	19.58 ± 1.66
MPH + HIIT	22.57 ± 0.66	19.51 ± 2.67
TM + MPH + HIIT	21.67 ± 1.06	21.48 ± 1.92

### 3.2 Effects of the MPH and HIIT on Passive Avoidance Following TM Administration

**FIGURE 4:**
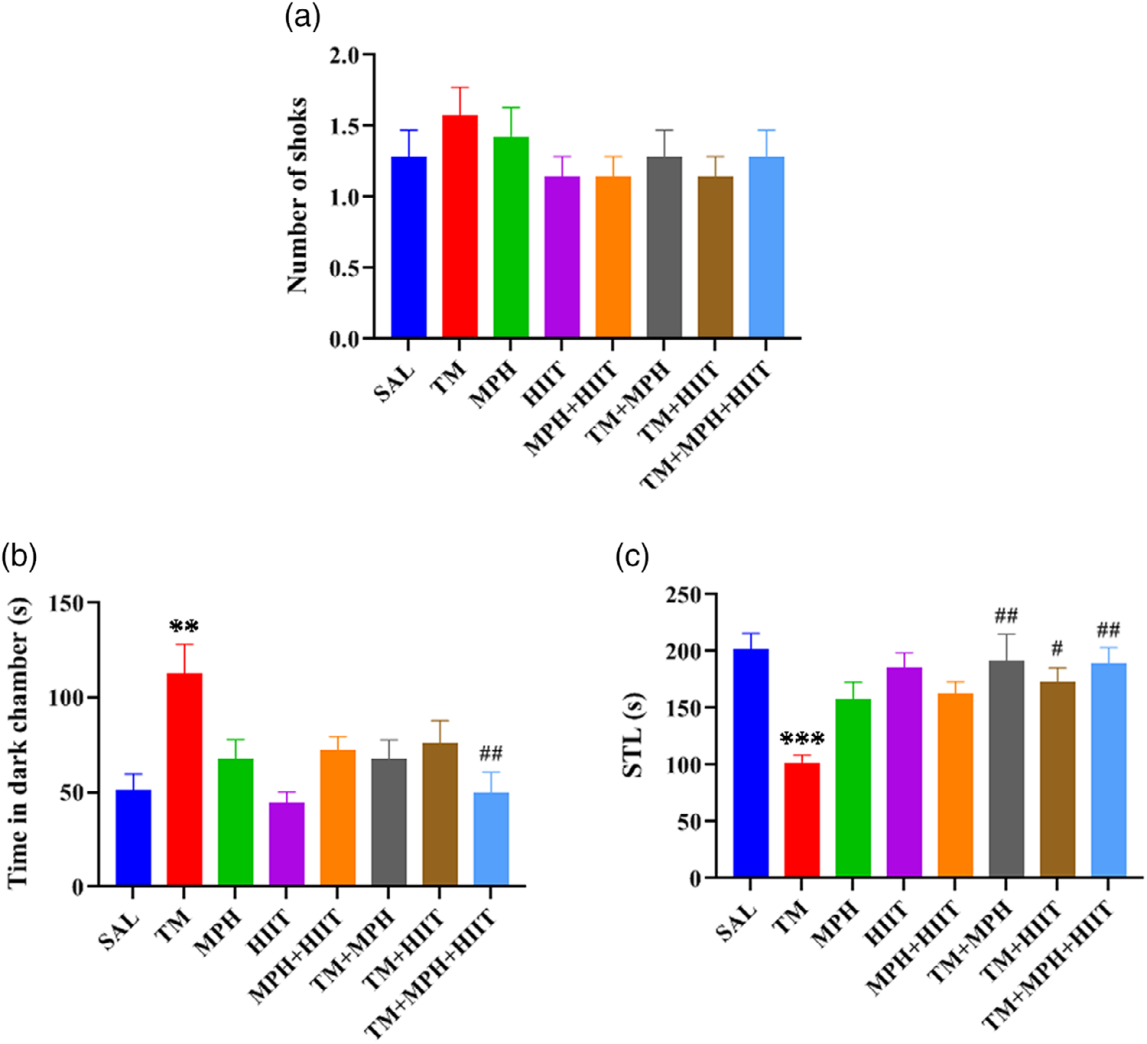
Passive avoidance performance. (A) No significant differences in shock number were observed between groups. (B) Time spent in the dark compartment increased in TM rats compared with SAL but decreased in TM + HIIT and TM + MPH + HIIT groups. (C) STL was reduced in TM compared with SAL but increased in the TM + MPH, TM + HIIT, and TM + MPH + HIIT groups. One‐way ANOVA. Data are mean ± SEM (*n* = 7). **p < 0.01, ***p < 0.001 versus SAL; ^#^ *p* < 0.05, ^##^ *p* < 0.01 versus TM.

The learning phase (shock number) showed no significant differences between groups, indicating no impairment in acquisition (Figure 4A).

In the memory phase, however, TM rats exhibited impairments, spending more time in the dark compartment (Figure 4B, *p* < 0.01) and showing reduced STL (Figure 4C, *p* < 0.001) compared with SAL. MPH improved memory in tramadol‐exposed rats by increasing STL in TM + MPH compared with TM (*p* < 0.01). Similarly, HIIT increased STL in TM + HIIT compared with TM (*p* < 0.05). In addition, a combination of HIIT and methylphenidate was able to decrease time spent in the dark compartment in the TM + MPH + HIIT group (Figure 4A; *p* < 0.01) and increase STL in the TM + MPH + HIIT group (Figure 4B; *p* < 0.01) compared to the TM group.

### 3.3 The Effects of the MPH and HIIT on the Oxidative Status Following TM Administration in the Hippocampus

**FIGURE 5:**
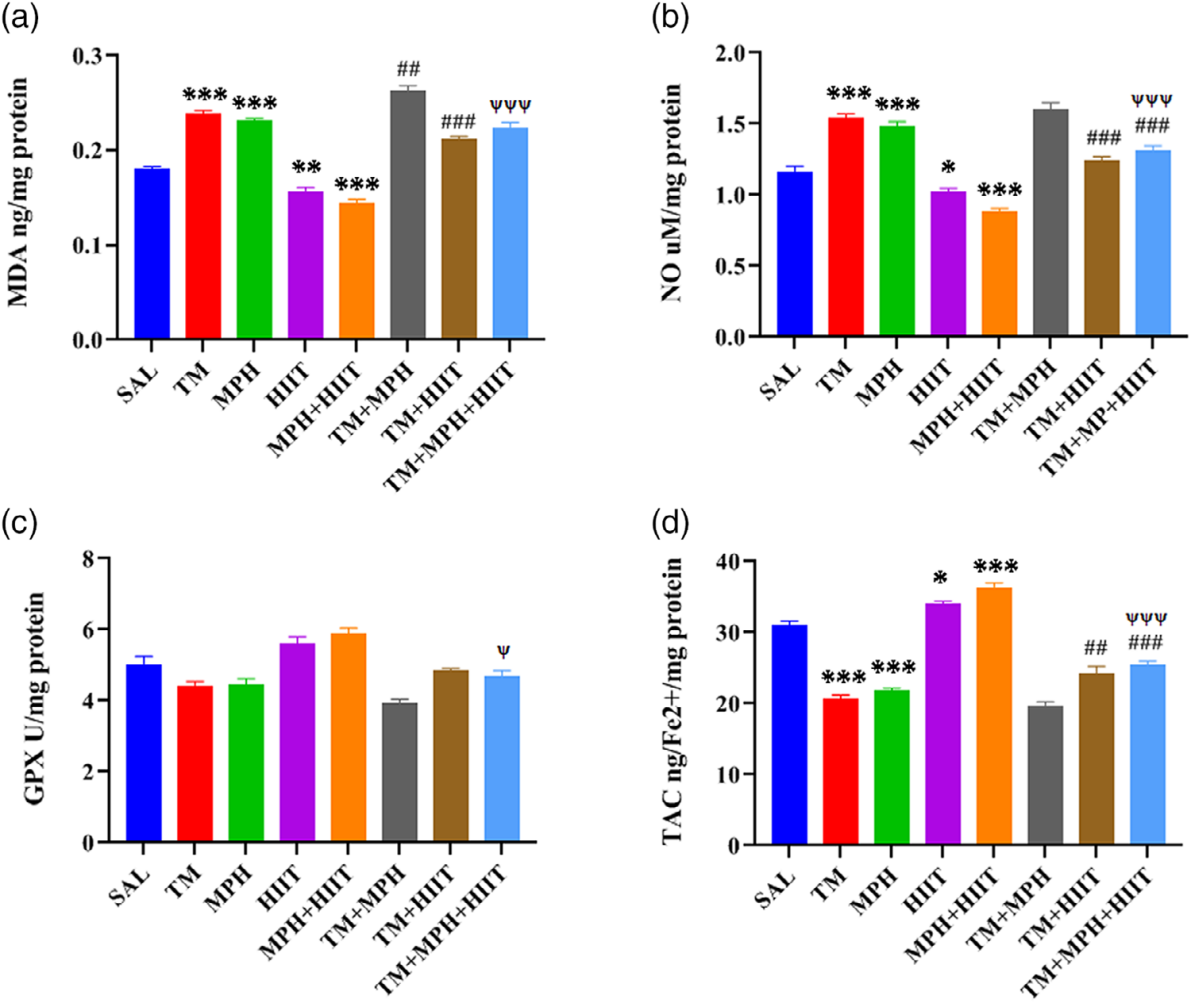
Effects of MPH and HIIT on hippocampal oxidative stress parameters: (A) MDA, (B) NO, (C) GPx, and (D) TAC. Tramadol and MPH increased MDA and NO and decreased TAC, whereas HIIT reduced MDA and NO and increased TAC. HIIT could reverse GPx level after combination with tramadol and methylphenidate. Tramadol and methylphenidate decreased TAC levels, and HIIT could increase TAC. One‐way ANOVA. Data are mean ± SEM (*n* = 4). The data are reported as mean ± SEM. *n* = 4. *p < 0.05, **p < 0.01, ***p < 0.001 versus SAL, ^##^ *p* < 0.01, ^###^ *p* < 0.001 versus TM, ^ψ^ *p* < 0.05, ^ψψψ^ *p* < 0.001 versus TM + MPH.

Tramadol significantly increased MDA (Figure 5A, *p* < 0.001) and NO (Figure 5B, *p* < 0.001) levels and decreased TAC (Figure 5D, *p* < 0.001) compared with SAL. MPH produced similar effects, elevating MDA and NO while lowering TAC compared with SAL (Figure 5A,B,D; *p* < 0.001).

HIIT reduced MDA (Figure 5A, *p* < 0.01) and NO (Figure 5B, *p* < 0.05) and increased TAC (Figure 5D, *p* < 0.05) compared with SAL. In tramadol‐exposed rats, HIIT reversed oxidative stress, reducing MDA (*p* < 0.001) and NO (*p* < 0.001) and increasing TAC (*p* < 0.01) compared with TM.

Furthermore, the combination of HIIT and methylphenidate decreased NO (Figures 5B, *p* < 0.001) and increased TAC (Figures 5D, *p* < 0.001) in the TM + MPH + HIIT group compared to the TM group.

In a comparison between the TM + MPH + HIIT and TM + MPH groups, our findings revealed that HIIT reversed the negative effects of methylphenidate and tramadol on oxidative stress parameters in the hippocampus. Specifically, HIIT significantly reduced MDA and NO levels (Figure 5A; *p* < 0.001 and Figure 5B; *p* < 0.001) and increased GPx and TAC levels (Figure 5C; *p* < 0.05 and Figure 5D; *p* < 0.001) in the TM + MPH + HIIT group compared to the TM + MPH group.

## 4 Discussion

This study investigated, for the first time, the effects of HIIT, MPH, and their combination on learning, memory, and oxidative stress markers in tramadol‐exposed rats. We observed that chronic tramadol administration impaired learning and memory and increased oxidative stress. Both MPH and HIIT alleviated tramadol‐induced cognitive deficits, but their mechanisms appeared to differ. HIIT effectively modulated hippocampal oxidative stress, whereas MPH disrupted redox balance despite improving cognitive performance.

Spatial learning and memory were assessed using the MWM. Our findings revealed that tramadol administration induced spatial learning and memory impairments in animals. Specifically, tramadol increased the total distance traveled and escape latency to find the hidden platform during the learning phase. It also decreased the distance traveled and time spent in the target quadrant during the memory phase of the MWM. Our findings are consistent with previous studies that reported tramadol exposure is associated with cognitive impairment, such as learning and memory deficits (Nafea et al. 2016; Baghishani et al. 2018).

The passive avoidance test is a fear‐motivated task utilized to assess learning and memory in rodents. In this test, animals learn to avoid an environment where an aversive stimulus (such as a foot shock) is delivered. Our results demonstrated that TM administration increased the time spent in the dark compartment and reduced the STL in the passive avoidance test in rats. Our findings support previous studies that have demonstrated that long‐term consumption of TM leads to cognitive impairments, such as deficits in passive avoidance memory (Baghishani et al. 2018; Jafari‐Sabet et al. 2016). Baghishani et al. (2018) showed that both the delay in entering the dark and the total time spent in the light compartment decreased in tramadol‐consuming rats.

The hippocampus plays a vital role in memory and in transferring information from short‐term to long‐term memory (Loureiro et al. 2012). Tramadol probably induces learning and memory impairments through several mechanisms, including elevated hippocampal apoptosis (Baghishani et al. 2018), neuroinflammation (Mowaad et al. 2023), and decreased levels of acetylcholinesterase (AChE) and monoamine oxidase (MAO) in the hippocampus (Mowaad et al. 2023).

Based on our findings, MPH attenuated the spatial learning and memory deficits induced by chronic tramadol administration, as it decreased escape latency to find the hidden platform in the learning phase and also enhanced distance traveled and time percent in the target quadrant during the memory phase. Our results support previous studies that revealed a positive effect of MPH on spatial memory (Motamedi et al. 2021; Khalid et al. 2020). Furthermore, MPH improved tramadol‐induced memory deficits in the passive avoidance task by increasing STL. Studies have suggested that MPH enhances synaptic transmission, ultimately improving memory (Khalid et al. 2020).

Evidence demonstrates that methylphenidate increases dopamine and norepinephrine signaling in the brain (Berridge et al. 2006), and both neurotransmitters regulate BDNF expression in the hippocampus (Mello‐Carpes et al. 2016; Morella et al. 2020), which has an important role in long‐term memory formation (Pastalkova et al. 2006). Methylphenidate could improve memory deficits through mechanisms including increased acetylcholinesterase activity (Scherer et al. 2010), enhanced synaptic gene expression, and decreased hippocampal inflammation (Khalid et al. 2020).

Several investigations have revealed the positive effects of exercise on learning and memory in humans and animals (Khodadadi et al. 2018; Yin et al. 2013; Mu et al. 2022; Zang et al. 2023; Cassilhas et al. 2016). Roig et al. (2013) reported the beneficial impacts of acute, but not long‐term, exercise on memory improvement in a time‐dependent manner by priming molecular processes involved in encoding and consolidating newly acquired data. Exercise performed 4 h before testing was associated with improved memory retention, suggesting that long‐term memory is enhanced following physical exercise at an appropriate time (van Dongen et al. 2016). Our findings support previous studies and reveal that 8 weeks of HIIT, like other forms of exercise, can improve learning and memory impairments in tramadol‐exposed rats, as it reduced total distance and escape latency to find the hidden platform while increasing the distance traveled and time spent in the target quadrant in the memory phase of the MWM. Additionally, our findings indicate that HIIT improved tramadol‐induced memory deficits in the passive avoidance task by increasing STL. Consistent with our results, many studies demonstrated the beneficial effects of HIIT on learning and memory (M. C. Lee et al. 2018; Gholipour et al. 2022). Treadmill exercise enhances passive avoidance memory by downregulating serotonin in the limbic system (Chen et al. 2008).

BDNF release is a crucial mechanism underlying the improvement of cognitive function after exercise (Erickson et al. 2019; Walsh et al. 2020). Exercise influences the function of BDNF and its specific receptor, tropomyosin receptor kinase B (TrkB), which are critically involved in synaptic plasticity and in learning and memory processes (Xu et al. 2021; Ahmadalipour et al. 2018). Previous studies have shown that physical exercise enhances neurogenesis and improves memory (S.‐E. Kim et al. 2010). Moreover, exercise can ameliorate memory by increasing norepinephrine and dopamine release into the synaptic cleft (Veening and Barendregt 2015) and increasing the number of new hippocampal cells (Biedermann et al. 2016). Furthermore, clinical studies have revealed that after a 6‐week exergaming training, the volumes of CA1, CA4, and DG were significantly increased in the left hippocampus of patients with Parkinson's disease (Schaeffer et al. 2022). Pre‐exposure to physical exercise markedly mitigated learning and memory impairments through its positive role in brain mitochondrial function (Mehdizadeh et al. 2017).

We also investigated oxidative stress parameters in the hippocampus of rats as a possible mechanism. Based on our results, tramadol disrupted hippocampal redox balance in rats. We observed a significant increase in MDA and NO levels and a significant reduction in TAC level in the hippocampus compared to saline‐treated rats. In addition, methylphenidate disrupted the hippocampal redox balance, similar to tramadol. Interestingly, HIIT modulated oxidative stress parameters, including a significant decrease in MDA and NO levels and a remarkable increase in TAC level in the rat's hippocampus. Our results showed that the simultaneous application of HIIT and MPH (i.e., the HIIT + MPH + TM group) could reverse the changes in oxidative stress parameters caused by the administration of MPH and TM in rats. HIIT seems to have enhanced the antioxidant system and prepared the body to counteract the potential oxidative stress induced by MPH. Therefore, MPH alone had no apparent beneficial effect on oxidative stress, but in combination with HIIT, the enhancing effect of HIIT on the antioxidant system was evident.

Our results support previous studies that determined TM‐induced memory impairments in rats are related to elevated oxidative stress (Khatmi et al. 2022; Ishola et al. 2022). Several studies have reported that physical exercise improves memory in rats, likely by reducing oxidative stress (Molaei et al. 2020; Jeong et al. 2021). Additionally, Albrakati (2020) showed that exercise significantly reduced TM‐induced neuronal apoptosis in the cerebral cortex of rats by suppressing oxidative stress.

Previous studies have revealed that MPH, similar to tramadol in some aspects, can cause oxidative and inflammatory alterations, leading to neuronal damage, particularly the degeneration of dopaminergic neurons (Motaghinejad, Motevalian, Shabab, et al. 2017; Thomas et al. 2004). Several studies have shown that chronic administration of MPH increases oxidative stress in the hippocampus (Motaghinejad, Motevalian, Babalouei, et al. 2017; Schmitz et al. 2012). However, our results demonstrated that MPH improved cognitive function in the MPH‐treated group, despite disrupting the redox balance. This suggests that MPH may improve cognitive functions through other mechanisms in the brain (Khalid et al. 2020; Scherer et al. 2010; Oakes et al. 2019), which requires further investigation.

MPH acts directly as an inhibitor of the dopamine transporter (DAT, SLC6A3) and the norepinephrine transporter (NET, SLC6A2) (Rajala et al. 2020). Thus, MPH is a dopamine and norepinephrine reuptake inhibitor (Shellenberg et al. 2020). It works by increasing the levels of monoamines (such as dopamine and norepinephrine) in the presynaptic cleft of several brain regions (Leary et al. 2017), ultimately leading to improved cognitive performance.

Additionally, MPH is considered an agonist for the 5‐hydroxytryptamine (serotonin) receptor 1A (HTR1A) (Markowitz et al. 2009). Since all these primary targets are involved in presynaptic signaling, MPH plays a role in regulating three main neurotransmitter (NT) pathways: dopamine (DA), norepinephrine (NE), and serotonin (5‐HT). Beyond these direct targets, MPH has been reported to indirectly modulate other proteins. This includes the stimulation of other NT pathways, such as adrenergic, dopaminergic, and glutamatergic receptors. This boost in neurotransmitters activates downstream pathways that are fundamental for synaptic plasticity and long‐term memory formation, even under conditions of mild oxidative stress (Zhang et al. 2012; Motaghinejad, Motevalian, Fatima, et al. 2017).

MPH also modulates neurotrophic factors involved in neuronal survival and plasticity, such as BDNF and its receptor, tropomyosin receptor kinase B (TrkB, also known as NTRK2) (Amiri et al. 2013). Furthermore, by modulating proteins like Bcl‐2 (BCL2), BAX, and caspase‐3 (CASP3), MPH can regulate the process of apoptosis, which also impacts neuronal survival (Réus et al. 2014). Finally, MPH has been reported to modulate intracellular mediators and transcription factors involved in neuronal signaling, including adenylate kinase isoenzyme 1 (AK1) and mitogen‐activated protein kinase 3 (MAPK3) (Coelho‐Santos et al. 2016).

Our results showed that tramadol probably induces cognitive impairment, while HIIT improves these deficits by reducing oxidative stress in the brain. Studies have demonstrated that methylphenidate improves cognitive function by preventing the reuptake of dopamine and norepinephrine in the synaptic cleft (Oakes et al. 2019). Clinical studies have reported that methylphenidate increases BDNF plasma concentrations in children with attention deficit hyperactivity disorder (ADHD) (Amiri et al. 2013). Methylphenidate can modulate apoptosis‐related proteins, including Bcl‐2 (BCL2), BAX, and caspase‐3 (CASP3) (Réus et al. 2014). Methylphenidate indirectly leads to the activation of postsynaptic dopamine receptors (Ko et al. 2019), which, upon binding dopamine, transmit the signal in postsynaptic neurons (Gronier 2011).

HIIT seems to be an effective treatment for cognitive impairment, especially following tramadol administration in rats, because it can act on brain function through several mechanisms, including reducing apoptosis. Treadmill exercise has been shown to suppress caspase‐3 expression in the hippocampus (J.‐H. Choi et al. 2013; B.‐K. Kim et al. 2014), increase B‐cell lymphoma 2 (Bcl‐2), and decrease Bcl‐2‐associated X (BAX) expression (Park et al. 2020). Furthermore, long‐term treadmill exercise improves spatial memory performance by decreasing tumor necrosis factor alpha (TNF‐α), interleukin‐6 (IL‐6), and interleukin‐1β (IL‐1β) (S.‐H. Kim et al. 2021). Exercise has also been shown to increase dendritic complexity and the number of dendritic spines in the dentate gyrus, leading to better cognitive performance (C.‐C. Lee et al. 2021). Meanwhile, Li et al. (2021) reported that adaptive treadmill training for 12 weeks, 5 days/week, 45 min a day, led to improved spatial learning and memory in mice, with significant increases in the number of synapses in the CA1 region of the hippocampus.

Based on the above findings and our results, it seems that HIIT could be considered an effective non‐pharmacological treatment for cognitive impairments induced by tramadol. It is recommended that future studies investigate other mechanisms through which HIIT can improve learning and memory in these animals.

This study has several limitations that should be addressed in future research. Although our oxidative stress data demonstrate a protective effect, further investigation is required to elucidate the upstream and downstream neurotrophic and cellular survival pathways involved. Due to financial constraints, the role of other brain regions and systems remains unclear. Additional research, particularly at the molecular and histological levels, is necessary to better understand the underlying mechanisms of the beneficial effects of HIIT in the context of tramadol administration.

## 5 Conclusion

Both MPH and HIIT improved tramadol‐induced cognitive impairments. HIIT improves these disorders, probably through modulating oxidative stress in the hippocampus. Although MPH disrupted the balance of oxidative stress, it probably improved cognitive impairments through another mechanism, which needs further molecular and histological investigations in future studies. Overall, it seems that HIIT can be an effective non‐pharmacological treatment for cognitive impairments in tramadol consumers.

## Author Contributions

**Sara Shirazpour**: methodology, writing – original draft. **Farahnaz Taheri**: writing – original draft, formal analysis, writing – review and editing. **Gholamreza Sepehri**: writing – original draft, supervision. **Manzumeh Shamsi Meymandi**: methodology. **Mahla Zangiabadizadeh**: methodology. **Mostafa Zangiabadi**: methodology. **Najmeh Sadat Hosseini**: methodology. **Mohammad Amin Rajizadeh**: writing – review and editing. **Sara Sheikhi**: methodology. **Nazanin Sabet**: methodology.

## Ethics Statement

All experimental procedures adhered to established ethical guidelines for the care and use of laboratory animals and were approved by the Institutional Animal Research Ethics Committee of Kerman University of Medical Sciences (Ethics code: IR.KMU.AEC.1401.005).

## Conflicts of Interest

The authors declare no conflicts of interest.

## Peer Review

The peer review history for this article is available at https://publons.com/publon/10.1002/brb3.70925.

## Data Availability

The data that support the finding of this study are available from the corresponding author upon reasonable request.
